# Preliminary experience of oblique occlusion technique in robot-assisted infrahepatic inferior vena cava thrombectomy: step-by-step procedures and short term outcomes

**DOI:** 10.1186/s12893-022-01821-7

**Published:** 2022-11-04

**Authors:** Zhuo Liu, Yuxuan Li, Shiying Tang, Xun Zhao, Kewei Chen, Liyuan Ge, Guodong Zhu, Peng Hong, Bingjun Wu, Zhiying Wu, Shudong Zhang, Xiaojun Tian, Shumin Wang, Cheng Liu, Hongxian Zhang, Lulin Ma

**Affiliations:** 1 Department of Urology, Peking University Third Hospital, Haidian District, 49 North Garden Road, Haidian, Beijing, 100191 People’s Republic of China; 2 Department of Ultrasound, Peking University Third Hospital, Haidian District, Beijing, 100191 People’s Republic of China

**Keywords:** Robotics, Laparoscopy approach, Renal cancer

## Abstract

**Background:**

We aimed to compare the oncological outcomes between the oblique occlusion technique and the traditional technique for robot-assisted radical nephrectomy (RARN) with inferior vena cava (IVC) thrombectomy, and to explore the safety and effectiveness of the oblique occlusion technique.

**Methods:**

Overall, 21 patients with renal cell carcinoma (RCC) and IVC tumor thrombus (TT) were admitted to our hospital from August 2019 to June 2020. All the patients underwent RARN with IVC thrombectomy, of which the IVC oblique occlusion technique was used in 11 patients and traditional occlusion technique was used in 10 patients. The oblique occlusion technique refers to oblique blocking from the upper corner of the right renal vein to the lower corner of the left renal vein using a vessel tourniquet or a vessel clamp (left RCC with IVCTT as an example).

**Results:**

Compared with patients in the traditional group, those in the oblique group had lower serum creatinine at follow-up (3 month) (95 ± 21.1 vs. 131 ± 30.7 μmol/L, P = 0.03). There was no significant difference in operation time [149 (IQR 143–245) min vs. 148 (IQR 108–261) min, p = 0.86], IVC clamping time [18 (IQR 12–20) min vs. 20 (IQR 14–23) min, p = 0.41], and estimated intraoperative blood loss [300 (IQR 100–800) mL vs. 500 (IQR 175–738) mL, p = 0.51] between both groups. During a 16-month (range, 15–23 months) follow-up period, two cases progressed in the oblique group and three cases progressed in the traditional group.

**Conclusions:**

The modified IVC oblique occlusion technique procedure is relatively safe and effective in RARN with IVC thrombectomy. The IVC oblique occlusion technique may play a role in the protection of renal function.

## Introduction

Renal cell carcinoma (RCC) is a common malignant tumor in the urinary system, and 4–10% of patients with locally advanced RCC have associated complication of inferior vena cava (IVC) tumor thrombus [1]. Surgical treatment is a traditional and effective treatment for RCC with inferior vena cava tumor thrombus (IVCTT) [2, 3]. With the widespread application of minimally invasive technology in urological tumors, many centers currently perform robot-assisted radical nephrectomy (RARN) with IVC thrombectomy [4–10]. In the classic surgical procedures of infrahepatic IVCTT, the vascular clamping technique is important in controlling hemorrhage during thrombectomy [7, 11]. It is usually necessary to clamp the caudal IVC, the contralateral renal vein (right renal artery should be clamped simultaneously for the left RCC with TT), and the cephalic IVC. This traditional approach ensures that the operation area is bloodless when the IVC wall is cut and the tumor thrombus is removed. However, this operation is associated with inevitable drawbacks, such as increased contralateral renal ischemia time [12]. In this study, we introduced an IVC oblique occlusion technique. The oblique occlusion technique refers to oblique blocking from the upper corner of the right renal vein to the lower corner of the left renal vein using a vessel tourniquet or a vessel clamp (left RCC with IVCTT as an example). We aimed to introduce the surgical procedures in a stepwise manner, and to explore the safety and effectiveness of the IVC oblique occlusion technique for RARN with IVC thrombectomy.

## Patients and methods

### Patients

We retrospectively analyzed the clinicopathological data of 21 patients with RCC and IVCTT who were admitted to our hospital from August 2019 to June 2020. All procedures were performed in accordance with the principles of the declaration of Helsinki, and all patients provided written informed consent. All the patients underwent RARN with IVC thrombectomy, of which 11 patients underwent the IVC oblique occlusion technique and 10 patients underwent the traditional occlusion technique. Renal tumors were classified according to the 2017 8th edition of the AJCC cancer staging manual [13], while IVC tumor thrombus was classified according to the Mayo classification [14]. Clinical symptoms were defined as simple local symptoms (such as hematuria, low back pain, abdominal mass, lower limb edema, etc.), simple systemic symptoms (such as emaciation, fatigue, etc.) that were found only during physical examination without other obvious symptoms, or both the local and systemic symptoms.

Indications of the IVC oblique technique include the following: (1) Mayo I and above classification; (2) Preoperative imaging examination indicating that there was no filling defect in the contralateral renal vein and the caudal IVC (the IVC below the renal vein), tumor thrombus (uneven enhancement after enhanced scan) or non-tumor thrombus (no obvious enhancement after enhanced scan); and (3) intraoperative ultrasound showed that there was no tumor thrombus or bland thrombus in the contralateral renal vein and the caudal IVC.

**Fig. 1:**
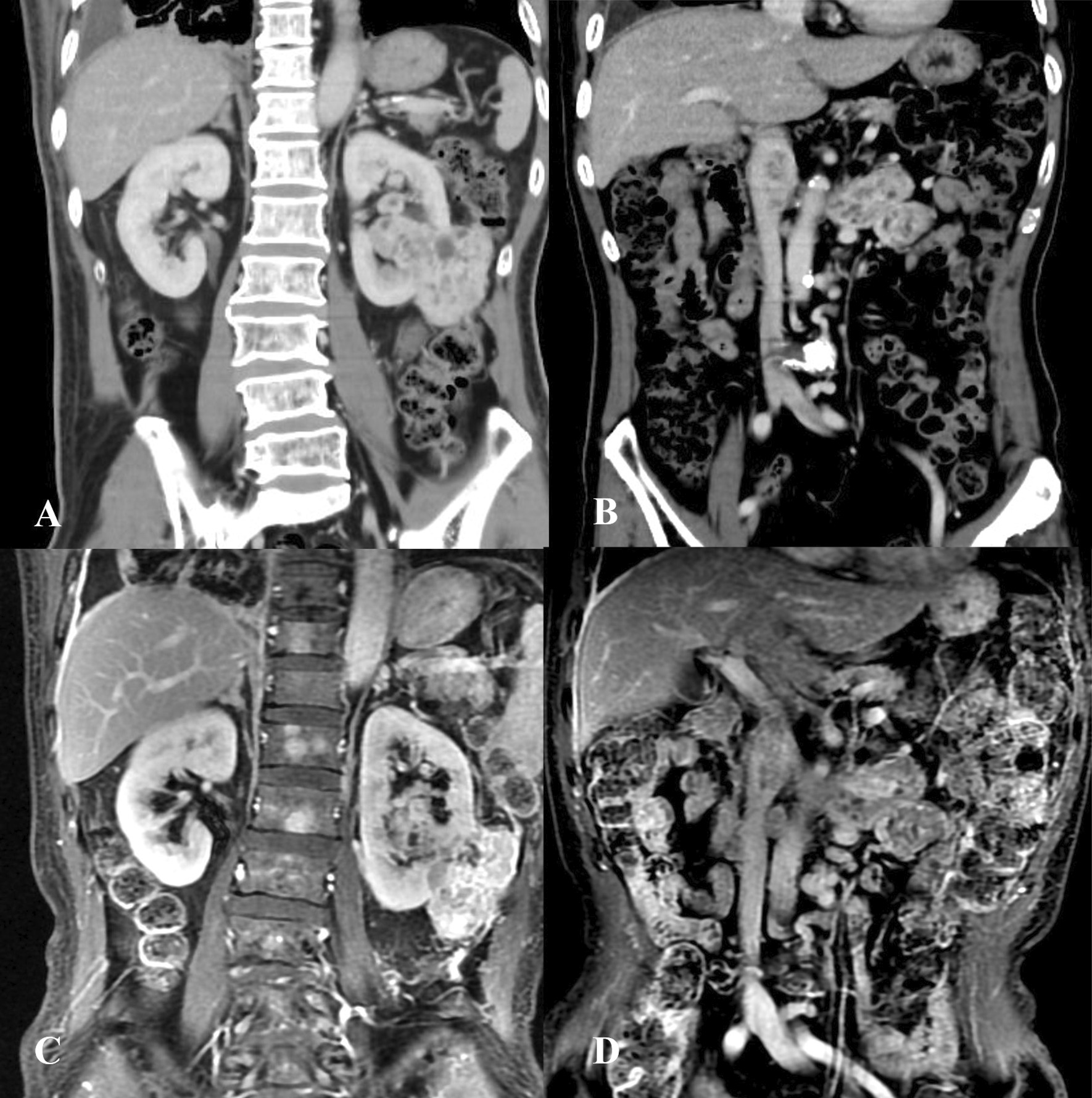
Preoperative imaging examination of a 72-year-old male patient. **A** Enhanced CT of the urinary system showed irregular soft tissue density of the left kidney in the range of 7.8 × 3.6 × 6.6 cm, and the enhanced scan showed uneven and obvious enhancement. Left renal cell carcinoma was considered for diagnosis. The tumor involved renal pelvis, calyces and perirenal fascia. **B** Soft tissue density was seen in the left renal vein and inferior vena cava, and uneven enhancement was showed on enhanced scan. The diagnosis considered tumor thrombus in the left renal vein and inferior vena cava. **C** Inferior vena cava enhanced magnetic resonance indicated left renal tumor. **D** Irregular filling defects were observed in the left renal vein and inferior vena cava, and the length of tumor thrombus in the inferior vena cava was 3.7 cm

All patients underwent enhanced computed tomography (CT) examination of the urinary system and enhanced magnetic resonance imaging (MRI) examination of IVC preoperatively to evaluate the side, size, and nature of the tumor, the length and maximum width of the tumor thrombus, presence of lymph node metastasis, adrenal invasion, liver metastasis, invasion of the IVC wall, bland thrombus (non-tumor thrombus), renal vein branch tumor thrombus, etc. (Fig. 1). All the patients were evaluated for distant metastasis using imaging examinations before surgery, including pulmonary CT, head MRI, or positron emission tomography computer tomography (PET-CT). The American Society of Anesthesiologists (ASA) score was used to evaluate the tolerance of patients to anesthesia.

### Preoperative preparation of patients

Patients were discussed by a multidisciplinary team before surgery. Routine bowel cleaning and skin preparation were performed 1 day preoperatively. Prophylactic second-generation cephalosporins or third-generation quinolone antibiotics were administered 30 min preoperatively. According to the characteristics of patients and the diseases, 400–2000 mL of suspended red blood cells were routinely prepared. A gastric tube was also inserted preoperatively.

### Surgical procedures

#### Oblique occlusion technique

**Fig. 2:**
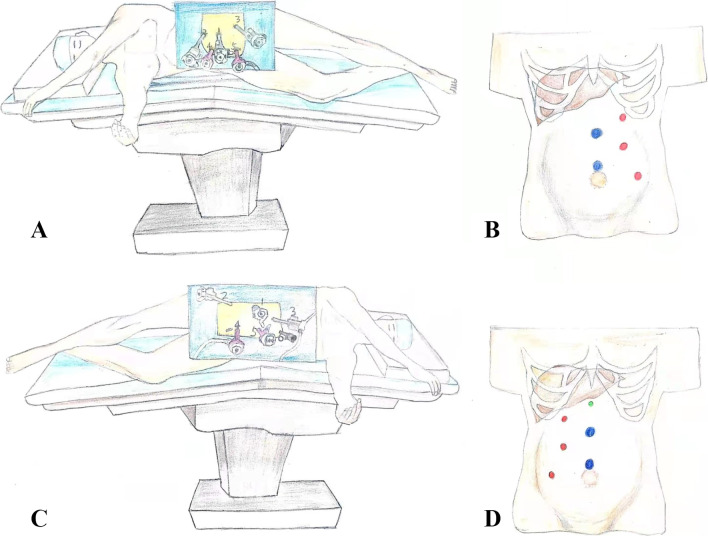
The surgical schematic showed the patient’s position and the placement of the trocar. For patients with left renal cell carcinoma with tumor thrombus. **A** Schematic diagram of patient position. The patient was placed in the right lateral decubitus position with chest pillow and tilted to the back by 30°. **B** Schematic diagram of the placement of the trocar. A 12-mm trocar (No. 1) was placed into the left lateral border of the rectus muscle under the 11th rib, and a laparoscope was placed. Under the direct laparoscopic vision, an 8-mm trocar (No. 2) was placed into the left lateral border of the rectus muscle under the costal margin for insertion of unipolar electric shear or needle holder. Another 8-mm trocar (No. 3) was placed on the inside of the iliac spine near the rectus muscle for inserting Maryland bipolar coagulation forceps, window grasping forceps, etc. A 12-mm trocar (No. 4) was placed around the umbilicus in the anterior midline, and another 12-mm trocar (No. 5) was placed 8 cm above the umbilicus. The auxiliary hole is used to insert a laparoscopic vascular clamp, suction, or serrefine. **C** Schematic diagram of the patient's position after changing the position. Patients were then repositioned in the left lateral decubitus position with chest pillow and tilted to the back by 30°. **D** Schematic diagram of the placement of the trocar after changing the position. An 8-mm trocar was placed into the right lateral border of the rectus muscle under the 11th rib, and a laparoscope was placed. Under the direct laparoscopic vision, two 8-mm trocars were placed into the right lateral border of the rectus muscle under the costal margin and on the inside of the iliac spine near the rectus muscle, and a 5-mm trocar was placed under the xiphoid

For cases of left RCC with IVCTT, the patient was placed in the right lateral decubitus position with chest pillows, and was tilted posteriorly by 30° (Fig. 2A). A veress needle was inserted around the umbilicus to establish a pneumoperitoneum. A 12-mm trocar (No. 1) was then placed into the left lateral border of the rectus muscle under the 11th rib; a laparoscope was also inserted. Under direct laparoscopic vision, an 8-mm trocar (No. 2) was placed into the left lateral border of the rectus muscle under the costal margin, and another 8-mm trocar (No. 3) was placed on the medial of the iliac spine near the rectus muscle. A 12-mm trocar (No. 4) was placed around the umbilicus in the anterior midline, and another 12-mm trocar (No. 5) was placed 8 cm above the umbilicus (Fig. 2B). The peritoneum was incised along the Toldt line outside the left paracolic sulcus. After the splenocolic, phrenicocolic, and splenorenal ligaments were disconnected, the splenic flexure of the colon was placed medially and cadually to reveal the left perirenal fascia. The left kidney was then removed from the perirenal fascia. The left renal vein was exposed and the tumor thrombus could be seen in the thickened vein. The left renal artery was dissociated and exposed under the renal vein, then clipped and cut off using a Hem-o-lok clip. The left adrenal vein, left gonadal vein and lumbar vein were dissociated and cut off. The left ureter was cut off at the lower pole of the left kidney. If there was no morphological change or tumor invasion, the left adrenal gland was preserved. After sufficient hemostasis, a the left perirenal drainage tube was inserted.

**Fig. 3:**
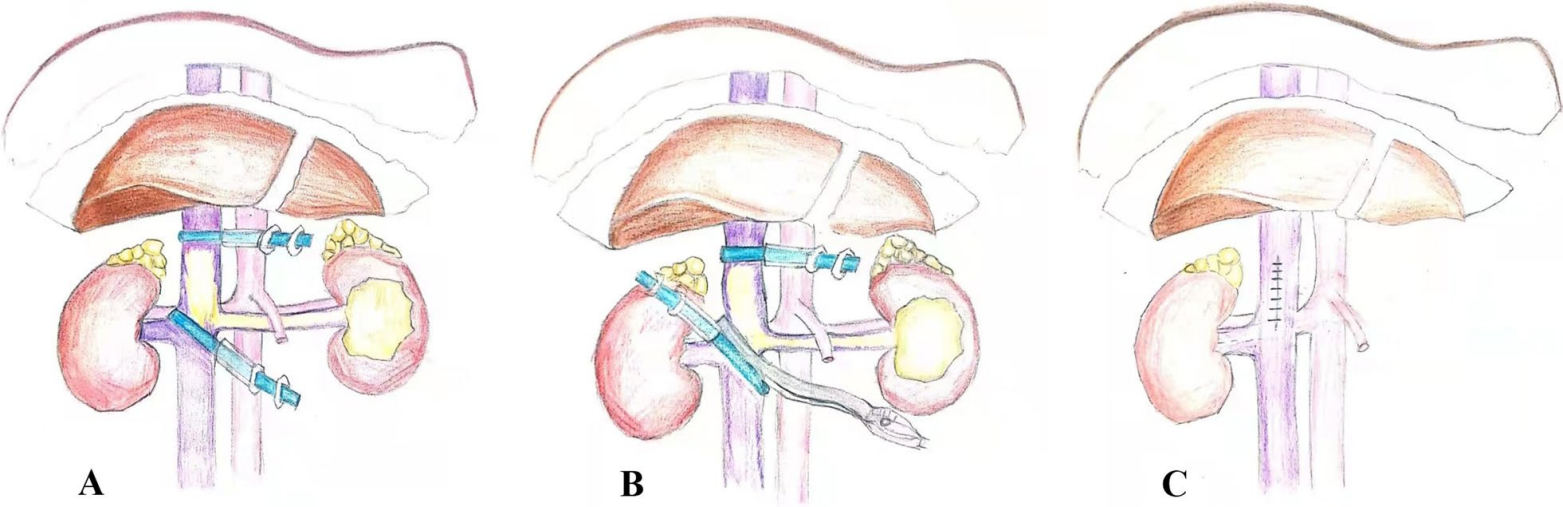
The surgical schematic shows the key steps of oblique occlusion. **A** Vessel tourniquet was placed in the cephalic inferior vena cava. Oblique blocking from the upper corner of the right renal vein to the lower corner of the left renal vein using a vessel tourniquet. **B** We occluded the caudal IVC using the oblique occlusion technique and then clamped the cephalic IVC. Oblique occlusion technique refers to oblique blocking from the upper corner of the right renal vein to the lower corner of the left renal vein using a vessel tourniquet and a vessel clamp. **C** After clipping the above vessels, the IVC wall was cut, and the thrombus was removed. After the IVC incision was continuously sutured by vascular line, the vessel tourniquet was released

**Fig. 4:**
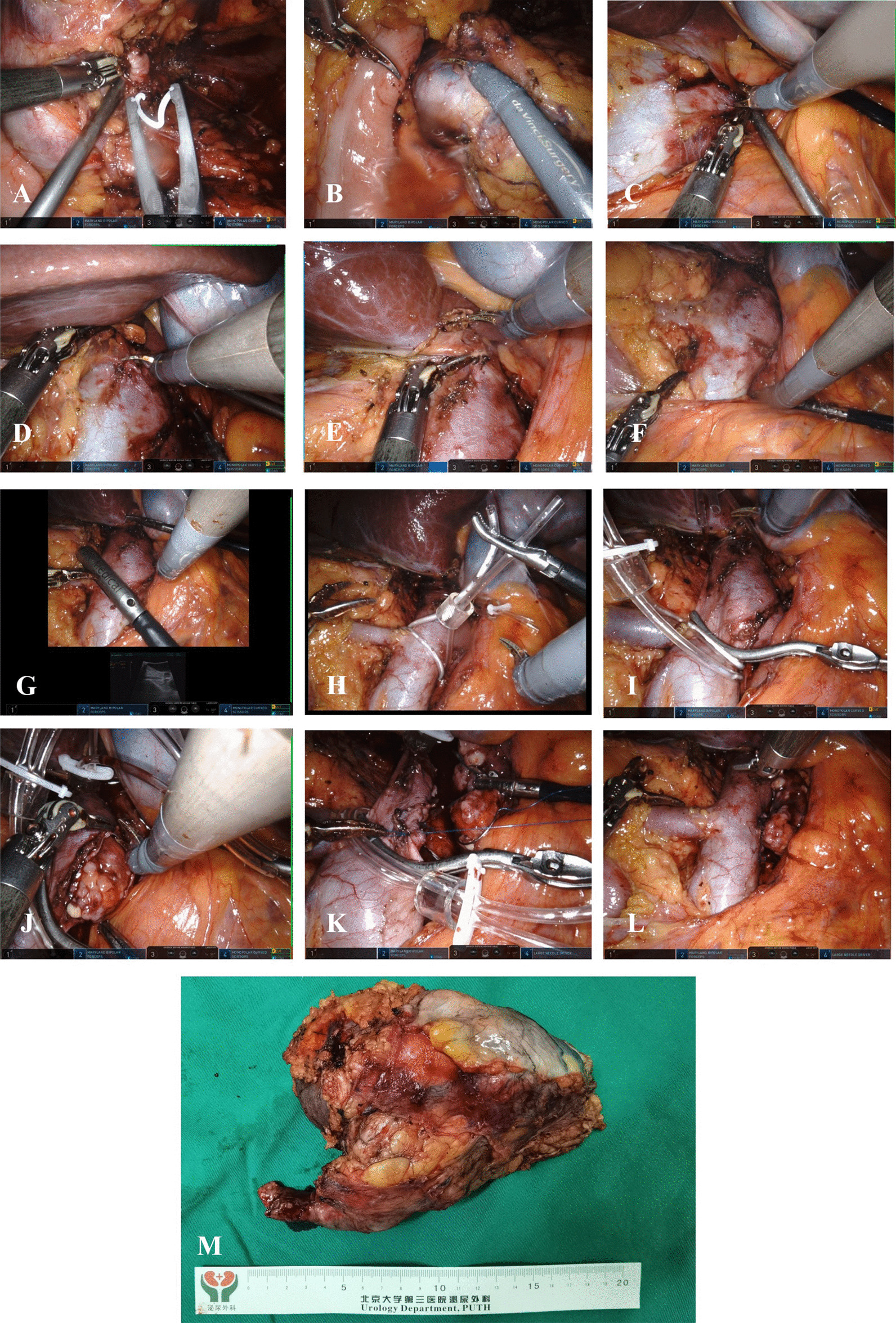
Step-by-step procedures of oblique occlusion technique in robot-assisted infrahepatic Inferior vena cava thrombectomy: **A** The left renal artery was clipped with a Hem-o-lok and cut off. **B** The left renal vein was exposed and tumor thrombus could be seen in the thickened vein. **C** The left renal vein was dissociated and exposed. **D** The cephalic inferior vena cava was dissociated and exposed. **E** Short hepatic vein was cut off. **F** The caudal IVC, the contralateral renal vein and the cephalic IVC was fully exposed. **G** Intraoperative ultrasound was used to detect the range of tumor thrombus to ensure that there was no tumor thrombus in the right renal vein and the IVC below the renal vein. **H** Oblique blocking from the upper corner of the right renal vein to the lower corner of the left renal vein using a vessel tourniquet or a vessel clamp. **I** The cephalic IVC was clamped. **J** The IVC wall was cut, and the thrombus was removed. **K** The IVC incision was sutured. **L** The vessel tourniquet was released. **M** Postoperative photos suggested: left renal cell carcinoma with inferior vena cava tumor thrombus

The patients were then repositioned in the left lateral decubitus position using chest pillow and tilted to posteriorly by 30° (Fig. 2C). An 8-mm trocar was inserted into the right lateral border of the rectus muscle under the 11th rib, and a laparoscope was inserted. Under the direct laparoscopic vision, two 8-mm trocars were placed into the right lateral border of the rectus muscle under the costal margin and on the inside of the iliac spine near the rectus muscle, and a 5-mm trocar was placed under the xiphoid process (Fig. 2D). The peritoneum was incised along the Toldt line outside the right paracolic sulcus. After the hepatocolic ligament and nephrocolic ligament were disconnected, the hepatic flexure of the colon was placed medially and caudally. The liver was retracted cephalically by inserting the needle holder from the trocar under the xiphoid process to clamp the abdominal wall. The right perirenal fascia was then cut and the duodenum was pushed medially. The IVC and right renal vein were dissociated and exposed, and the left renal vein was further dissociated. Intraoperative ultrasound was used to detect the range of tumor thrombus to ensure that there was no tumor thrombus in the right renal vein and the IVC below the renal vein. We occluded the caudal IVC using the oblique occlusion technique and then clamped the cephalic IVC (Fig. 3). The oblique occlusion technique refers to oblique blocking from the upper corner of the right renal vein to the lower corner of the left renal vein using a vessel tourniquet or a vessel clamp. After clipping the above vessels, the IVC wall was cut at the angle between the left renal vein and the IVC, and the thrombus was removed. If a tumor thrombus was found to be adherent to the IVC wall, it is necessary to remove the invaded vascular wall and suture the incision. After the IVC incision was continuously sutured using a vascular line, the vessel tourniquet was released (Fig. 4). The IVC clamping time started when the IVC was clamped using the vascular blocking clamp, and ended after the vascular blocking clamp was released. Finally, the IVCTT and left kidney specimens were removed through a median abdominal median incision.

Regarding cases of right RCC with IVCTT, the patient was placed in the left lateral decubitus position, and the placement of trocars were the same as for cases of left RCC with IVCTT. The steps were similar except for the obliquely blocked blood vessel. For cases of right RCC with IVCTT, we used vessel tourniquet or vessel clamps to obliquely block blood flow from the upper corner of the left renal vein to the lower corner of the right renal vein.

The procedures for addressing bleeding if the clamps were dislodged prior to opening the vena cava are as follows: The operation should be as gentle as possible to avoid violent movements, so as to reduce the risk of blocking clamps displacement. If intraoperative vascular clamp displacement causes bleeding, the pneumoperitoneum pressure can be increased appropriately to prevent IVC hemorrhage. In previous studies, we systematically introduced how to improve pneumoperitoneum pressure to ensure that blood in the IVC but did not flow out of the IVC [15]. The source of bleeding is usually from the contralateral renal vein or the distal end of the IVC, which can be blocked using vascular clamp in the corresponding blood vessels to prevent bleeding. Moreover, in the initial stage of the application of this method, the vascular occlusion band can be reserved from the upper right renal vein to the lower left renal vein. If the vascular clamp is dislodged, the reserved vascular band can be tightened to prevent hemorrhage. In addition, the surgeon should be calm when dealing with emergencies during the operation, and should avoid panic and be flexible according to the specific conditions during the operation.

The procedures for addressing bleeding if unsuspected venous tributaries are not identified prior to opening the vena cava are as follows: First, before the operation, an enhanced MRI examination of the IVC should be performed to determine whether small branch of the IVC are present. Furthermore, the distal, proximal, and contralateral renal veins of the IVC need to be fully exposed before vascular occlusion. To make the blocked area bloodless, all tributaries of the IVC under direct vision should be fully cut. For venous tributaries with small diameters, a bipolar coagulation device can be selected to coagulate them. Furthermore, intraoperative pneumoperitoneum pressure can be increased to prevent bleeding from the IVC.

#### Traditional occlusion technique

The patient's position and the placement of trocars in the traditional occlusion method were the same as those in the oblique occlusion group. However, compared to the oblique occlusion technique, in conventional IVC thrombectomy, it is usually necessary to clamp the caudal IVC, contralateral renal vein (the right renal artery should be clamped simultaneously for cases of left RCC), and cephalic IVC.

### Postoperative management of patients

Prophylactic anticoagulation was not used routinely after the operation because it may cause bleeding from wounds and increase the overall risk of postoperative bleeding. We encouraged patients to ambulate early. Those who could not get out of their beds were instructed to actively flex their lower limbs and perform ankle plantar flexion, dorsiflexion, and internal and external rotation, as well as wear elastic socks or elastic bandages. If the lower extremity deep venous thrombosis was clearly diagnosed, the patient was instructed to rest in bed and reduce massage or intense activity, to prevent the thrombosis from being propagated. Patients and staff were also instructed to pay close attention to lower limb edema, record the changes in lower limb circumference and pay attention to the circumferences of both the affected and the healthy sides. They were instructed to keep the affected limb warm and closely observe the changes in skin temperature. If patients were diagnosed with lower extremity deep vein thrombosis postoperatively, low molecular weight heparin could be injected subcutaneously and relevant coagulation indicators are reviewed regularly.

Postoperative complications were evaluated using the Clavien-Dindo classification method, and those with grade III or IV were defined as severe complications [16]. Follow-up was performed after 3 months, including survival status, postoperative renal function assessment, and tumor recurrence or metastasis.

### Statistical analysis

The normality of continuous variables was tested and normally distributed data were expressed as mean (standard deviation); independent samples T-test was used to analyze these data. Non-normally distributed data were expressed as median (IQR), and the Mann–Whitney U test was used to analyze such data. Categorical data were expressed as frequency (percentage), using chi-square test, or the Fisher's exact test was used if the chi-square test was not met, and p < 0.05 indicated that the difference was statistically significant. All statistical analyses were performed using SPSS version 18.0 (IBM, Armonk, NY, USA).

## Results

The baseline characteristics of the patients are listed in Table 1. All the 21 patients underwent RARN with IVC thrombectomy: the IVC oblique occlusion technique was used in 11 patients and the traditional occlusion technique was used in ten patients. There was no significant difference between the oblique and traditional group in terms of age, gender, body mass index (BMI), tumor side, clinical stage, IVC thrombus classification, IVC thrombus length, or clinical symptoms.

**Table 1:** Comparison of baseline characteristics between oblique and traditional technique in robot-assisted radical nephrectomy with IVC thrombectomy

Characteristics	Oblique (n = 11)	Traditional (n = 10)	P
Age (year), mean (SD)	56.1(14.1)	55.6(15.8)	0.94
*Gender, n*			1.00
Male	7	6	
Female	4	4	
BMI (kg/m^2^), mean (SD)	24.8(2.3)	27.2(3.9)	0.10
*Affected kidney, n*			0.67
Left	4	5	
Right	7	5	
Renal tumor size (cm), mean (SD)	5.8(2.5)	8.8(2.8)	0.02*
*Clinical stage, n*			0.22
cT3N0M0	4	5	
cT3N1M0	1	4	
cT3N0M1	2	0	
cT3N1M1	2	1	
cT4N1M1	2	0	
*IVC thrombus classification, n*			0.47
I	5	5	
II	5	3	
III	0	2	
IV	1	0	
IVC thrombus length (cm), mean (SD)	3.1(2.3)	3.4(2.5)	0.80
*Clinical symptoms, n*			0.66
No	5	2	
Local symptoms	5	7	
Systemic symptoms	1	1	
Both	0	0	

BMI, body mass index; IVC, inferior vena cava; SD, standard deviation

Data presented as mean (SD). *p < 0.05

A comparison of clinicopathological features between the oblique and traditional techniques is shown in Table 2. There was no significant difference between the oblique and traditional groups in terms of preoperative and postoperative blood urea nitrogen (BUN). The average preoperative serum creatinine in the oblique group and the traditional group were 73 ± 20.1 μmol/L and 96 ± 28.7 μmol/L, respectively, and the difference was not statistically significant (p = 0.06). While the average postoperative (1 week) serum creatinine of the oblique group and traditional groups were 98 ± 37.7 μmol/L and 120 ± 30.0 μmol/L, respectively, there was also no significant difference(p = 0.19). However, compared with the traditional group, oblique group had lower serum creatinine at follow-up (3 month) (95 ± 21.1 vs 131 ± 30.7 μmol/L, p = 0.03).

**Table 2:** Comparison of clinicopathological features between oblique and traditional technique in robot-assisted radical nephrectomy with IVC thrombectomy

Characteristics	Oblique	Traditional	p
Operative time (min), median (IQR)	149 (143–245)	148 (108–261)	0.86
IVC clamping time (min), median (IQR)	18 (12–20)	20 (14–23)	0.41
Estimated blood loss (ml), median (IQR)	300 (100–800)	500 (175–738)	0.51
Patients receiving transfusion, n (%)	4 (36.4)	5 (50)	0.67
Transfer to intensive care unit, n (%)	3 (27.3)	3 (30)	1.00
Days to surgical drain removal, median (IQR)	4 (3–5)	5 (4–7)	0.05
Days to full ambulation, median (IQR)	3 (3–3)	2 (2–3)	0.17
Days to oral feeding, median (IQR)	2 (2–3)	3 (2–6)	0.28
Postoperative hospital stay (days), median (IQR)	7 (5–10)	8 (5–9)	0.86
*ASA grade, n (%)*			0.21
Grade II	8 (72.7)	10 (100)	
Grade III	3 (27.3)	0	
*Postoperative complication, n (%)*			0.06
Grade I	4 (36.4)	0	
Grade II	2 (18.2)	6 (60)	
Grade III	0	0	
Grade IV	0	0	
Preoperative Hb (g/l), mean (SD)	135 (13.2)	117 (23.3)	0.05
Postoperative Hb (g/l), mean (SD)	110 (18.0)	104 (19.8)	0.50
Preoperative serum Cr (μmol/l), mean (SD)	73 (20.1)	96 (28.7)	0.06
Postoperative (1 week) serum Cr (μmol/l), mean (SD)	98 (37.7)	120 (30.0)	0.19
Serum Cr at follow-up (3 months; μmol/l), mean (SD)	95 (21.1)	131 (30.7)	0.03*
Preoperative serum BUN (mmol/l), mean (SD)	5.3 (1.2)	5.8 (2.3)	0.53
Postoperative (1 week) serum BUN (mmol/l), mean (SD)	4.6 (2.1)	6.3 (3.4)	0.19
Serum BUN at follow-up (3 months; mmol/l), mean (SD)	5.7 (1.3)	7.2 (2.5)	0.18
*Postoperative histology, n (%)*			0.33
ccRCC	9 (81.8)	5 (50)	
Papillary RCC	0	2 (20)	
Unclassified RCC	1 (9.1)	0	
Xp11.2 RCC	0	1 (10)	
Urothelial carcinoma	0	1 (10)	
Angiomyolipoma	1 (9.1)	1 (10)	
*Tumor grade, n (%)*			0.07
Grade II	5 (55.6)	1 (10)	
Grade III	1 (11.1)	5 (50)	
Grade IV	3 (33.3)	2 (20)	
Perirenal fat invasion, n (%)	2 (18.2)	1 (10)	1.00
IVC wall invasion, n (%)	0	5 (50)	0.01*
Presence of bland thrombus, n (%)	0	3 (30)	0.09
Presence of renal vein branch tumor thrombus, n (%)	0	1 (10)	0.48
Lymph node metastasis, n (%)	4 (36.4)	2 (20)	0.36
Distant metastasis, n (%)	6 (54.5)	1 (10)	0.06
Preoperative targeted therapy, n (%)	1 (9.1)	0	1.00
Adjuvant targeted therapy, n (%)	8 (72.7)	3 (30)	0.09

IQR, interquartile range; SD, standard deviation; ASA, American Society of Anesthesiologists; Hb, hemoglobin; Cr, creatinine; BUN, blood urea nitrogen; ccRCC, clear cell renal cell carcinoma; IVC, inferior vena cava

Data presented as mean (SD) or median (IQR), unless otherwise noted. *p < 0.05

Regarding the 11 patients who underwent the IVC oblique occlusion technique, the median operative time was 149 (IQR: 143–245) min and the median IVC clamping time was 18 (IQR: 12–20) min. The median estimated intraoperative blood loss was 300 (IQR: 100–800) mL, and four patients (36.4%) received blood transfusions. When these characteristics were compared with those of the traditional group, no significant difference was observed.

No patients had IVC wall invasion, bland thrombus, or renal vein branch tumor thrombus in the oblique group. However, in the traditional group, five (50%) patients had IVC wall invasion, three (30%) patients had a bland thrombus, and one (10%) patient had a renal vein branch tumor thrombus.

In the oblique group, six (54.5%) patients had postoperative complications; however, no severe complication occurred. Furthermore, four patients had mild renal dysfunction, one patient who had diarrhea was treated without anti-infective therapy, and one patient needed postoperative blood transfusion due to anemia. While in the traditional group, six (60%) patients had postoperative complications and no severe complication occurred. One patient had incomplete intestinal obstruction, one patient had lymphorrhagia, one patient had pancreatitis, and three patients needed postoperative blood transfusions due to anemia.

The median follow-up period in this study was 16 months (range, 15–23 months), two cases progressed (one bone metastasis and one adrenal metastasis) in the oblique group, while three cases progressed (one liver metastasis and two lung metastasis) in the traditional group.

## Discussion

In traditional tumor thrombus surgery, vascular occlusion is an essential surgical procedure during thrombectomy [17, 18]. Complete and adequate vascular occlusion is a significant prerequisite for subsequent surgical procedures. Before cutting the walls of the IVC with scissors, it is necessary to ensure that there is no blood flow in the IVC section, including the tumor thrombus. Incomplete occlusion of the tributaries of the IVC could lead to massive hemorrhage after the IVC wall is incised. Intraoperative hemorrhage could cause the patient's hemoglobin level to decrease, and could lead to low intraoperative blood pressure, circulatory instability, and even severe hemorrhagic shock. Moreover, bleeding affects surgical vision and causes technical difficulties in the subsequent steps of thrombectomy and suturing of the wall of the IVC, which will increase the difficulty of the operation, cause surgical stagnation, and seriously endanger the health and life of the patients. The oblique occlusion technique introduced in this study ensures the surgical requirement of no blood flow in the IVC and achieves the same surgical effect as traditional tumor thrombus surgery.

The right renal vein has few branches because most right adrenal and gonadal veins drain into the IVC directly. In contrast, the left renal vein has several tributaries. The branches of the left renal vein usually include the left adrenal vein, left gonadal vein and left ascending lumbar vein. For cases of right RCC and IVCTT, the reasons for blocking the left renal vein without blocking the left renal artery are as follows: the left renal artery is responsible for left renal blood perfusion, while the three main tributaries of and left renal vein are responsible for blood reflux. After clamping the left renal vein, blood flow from the left renal artery can still return through the tributaries of the left renal vein. Therefore, left renal artery occlusion is not routinely performed intraoperatively. For cases of left RCC and IVCTT, the reasons for clamping the right renal artery and vein simultaneously are as follows: if the right renal vein is occluded without the compensatory backflow of the branch vein and the right renal artery remains unobstructed, congestion could occur in the right kidney. Renal congestion can lead to renal dysfunction. Therefore, it is necessary to block both the right renal artery and vein. The oblique occlusion technique is particularly suitable for left RCC with IVCTT.

Traditionally, the corresponding vascular occlusion should be performed before the IVC wall is cut and the thrombus is removed. In conventional infrahepatic IVC thrombectomy, it is usually necessary to clamp the caudal IVC, contralateral renal vein (right renal artery should be clamped simultaneously for left RCC), and cephalic IVC. Although many studies have found that renal ischemia within 30 min does not significantly affect postoperative renal function [19], there are still risks. Cephalic IVC handling in the oblique occlusion method is the same as that in the traditional technique. The difference is that in the oblique occlusion method we did not clamp the contralateral renal artery and vein when the caudal IVC was treated. The specific surgical procedure is obliquely blocking from the upper corner of the right renal vein to the lower corner of the left renal vein using a vessel tourniquet and a vessel clamp (using cases of left RCC with IVCTT as examples). Vessel clamps can effectively prevent the opening of the contralateral renal vein and prevent the IVC from tearing. The advantages of the oblique blocking technique are as follows: first, it satisfies the surgical requirement of no blood flow in the IVC, and achieves the same surgical effect as traditional surgery. Second, the risk of ischemia in the contralateral kidney is decreased, as the technique ensures that right renal blood can return to the IVC through the unobstructed right renal vein. Simultaneously, it ensures that the contralateral kidney has adequate blood supply and reduces the probability of postoperative renal insufficiency. We found that the oblique occlusion technique could significantly improve postoperative renal function compared to the traditional technology technique. Serum Cr level at the 3-month follow-up visit in the oblique group was significantly different form that of the traditional group (95 μmol/L vs. 131 μmol/L, p = 0.03). Some scholars have shared their experience regarding whether it is feasible for the right renal vein to drain into the caudal IVC [11]. This method plays a vital role in protecting renal function in patients with isolated kidney after radical nephrectomy.

Reasonable indications for patient selection is the premise of safe and effective surgery. The oblique occlusion technique is more suitable for floating tumor thrombus, and more caution is needed for filled-type tumor thrombus. The clinical features of filled-type tumor thrombus were described in detail in our previous article [20]. The use of this method will be limited to cases of IVCTT or bland thrombus with caudal extension. Therefore, urinary enhanced CT and IVC enhanced MRI should be performed preoperatively. The oblique occlusion technique is not applicable in cases of severe filled-type tumor thrombus obstruction, tumor thrombus growth into the caudal IVC (below the renal vein opening), and when there are long segment bland thrombus in the caudal tumor thrombus. The surgical treatment of IVCTT complicated with distal bland thrombus has been introduced in detail in previous studies [21]. In addition, we have also introduced the need for segmental IVC resection or IVC transection in previous studies [22], which is not suitable for this method.

An essential auxiliary tool in oblique occlusion technique is the use of intraoperative ultrasound, which could help in determining the extent of the tumor thrombus [23]. For example, for cases of left RCC with IVCTT, intraoperative ultrasound can be used to determine whether there is a tumor thrombus at the opening of the right renal vein or inside it, and whether there is a tumor thrombus at the lower corner of both renal veins or the caudal IVC. Intraoperative ultrasound also ensures the safety of the oblique occlusion technique, which can prevent the vascular occlusion band from embedding into the tumor thrombus tissue, causing the tumor thrombus to propagate. In addition, intraoperative ultrasound can help to determine the height of the proximal tumor thrombus and ensure that the cephalic IVC vessel tourniquet is above the proximal tumor thrombus to ensure complete occlusion.

This study has some limitations. The oblique occlusion technique reduces the time of ischemia of the contralateral kidney owing to the principle of the procedure. Although the traditional occlusion method may theoretically cause postoperative renal insufficiency, it is challenging to detect renal dysfunction when the renal ischemia time is controlled within 30 min. Another limitation is the sample size. We did not strictly match these variables in the traditional and oblique groups. On the one hand, although an indication of the oblique occlusion technology was the absence of invasion of the IVC wall, bland thrombus, or tumor thrombus of the renal vein branch, these variables did not exist in the oblique group. Moreover, in our previous study, the involved vessel wall was removed in patients in which the invasion area of the IVC wall was small. The renal vein outflow was reserved to allow the healthy renal blood flow back into the IVC, which had minimal effects on postoperative renal function [20]. In another previous study [21], thrombectomy rather than IVC transection was still available for patients with short-segment IVC bland thrombus and had no significant effect on postoperative renal function. Patients with IVC wall invasion and bland thrombus in this study met the above classification. Therefore, we consider that these specific patients included in the traditional group did not significantly impact the results of this study.

## Conclusions

In conclusion, this modified IVC oblique occlusion technique procedure was relatively safe and effective in RARN with IVC thrombectomy. The IVC oblique occlusion technique may play a role in protecting renal function.

## Data Availability

The datasets used and/or analysed during the current study available from the corresponding author on reasonable request.
